# Adaptive Immune Responses in a Multiple Sclerosis Patient with Acute Varicella-Zoster Virus Reactivation during Treatment with Fingolimod

**DOI:** 10.3390/ijms160921832

**Published:** 2015-09-10

**Authors:** Andrea Harrer, Peter Wipfler, Georg Pilz, Katrin Oppermann, Elisabeth Haschke-Becher, Shahrzad Afazel, Jörg Kraus, Eugen Trinka, Johann Sellner

**Affiliations:** 1 Department of Neurology, Christian Doppler Medical Center, Paracelsus Medical University, 5020 Salzburg, Austria; E-Mails: a.harrer@salk.at (A.H.); p.wipfler@salk.at (P.W.); georg.pilz@salk.at (G.P.); k.oppermann@salk.at (K.O.); e.trinka@salk.at (E.T.); 2 Department of Laboratory Medicine, Paracelsus Medical University, 5020 Salzburg, Austria; E-Mails: e.haschke-becher@salk.at (E.H.-B.); s.afazel@salk.at (S.A.); 3 Department of Neurology, A.ö. Krankenhaus Zell am See, Teaching Hospital of the Paracelsus Medical University, 5700 Zell am See, Austria; E-Mail: joerg.kraus@kh-zellamsee.at; 4 Research Institute of Neurointervention, Paracelsus Medical University, 5020 Salzburg, Austria; 5 Department of Neurology, Klinikum rechts der Isar, Technische Universität, 81675 München, Germany

**Keywords:** VZV reactivation, fingolimod, treatment discontinuation, immune reconstitution, peripheral blood, cerebrospinal fluid

## Abstract

Fingolimod, an oral sphingosine 1-phosphate (S1P) receptor modulator, is approved for the treatment of relapsing forms of multiple sclerosis (MS). The interference with S1P signaling leads to retention particularly of chemokine receptor-7 (CCR7) expressing T cells in lymph nodes. The immunological basis of varicella zoster virus (VZV) infections during fingolimod treatment is unclear. Here, we studied the dynamics of systemic and intrathecal immune responses associated with symptomatic VZV reactivation including cessation of fingolimod and initiation of antiviral therapy. Key features in peripheral blood were an about two-fold increase of VZV-specific IgG at diagnosis of VZV reactivation as compared to the previous months, a relative enrichment of effector CD4+ T cells (36% *versus* mean 12% in controls), and an accelerated reconstitution of absolute lymphocytes counts including a normalized CD4+/CD8+ ratio and reappearance of CCR7+ T cells. In cerebrospinal fluid (CSF) the lymphocytic pleocytosis and CD4+/CD8+ ratios at diagnosis of reactivation and after nine days of fingolimod discontinuation remained unchanged. During this time CCR7+ T cells were not observed in CSF. Further research into fingolimod-associated VZV reactivation and immune reconstitution is mandatory to prevent morbidity and mortality associated with this potentially life-threatening condition.

## 1. Introduction

Varicella-zoster virus (VZV) is a neurotropic α-herpes virus which can cause clinically distinct presentations. Primary infection with VZV occurs naturally during childhood and is characterized by a self-limiting generalized vesicular rash. Reactivation of latent VZV infection in cells of the cranial nerve, dorsal root and autonomic ganglia comprises the second manifestation [1]. The corresponding mechanism of action is the assembly of new viral particles and transport in anterograde direction. VZV reactivation most commonly manifests as localized, unilateral, and painful vesicular skin infection. This condition can be complicated by post-herpetic neuralgia, meningoencephalitis, and vasculopathy [2]. VZV-specific T-cell responses, in particular CD8+ effector memory T cells are considered the major host defense against symptomatic VZV reactivation from latency [3]. This antigen-specific cellular immune response, however, declines with age or immunosuppression, and exposes this group of individuals to an increased risk of VZV reactivation.

Fingolimod (FTY720, Gilenya) is a sphingosine-1-phosphate (S1P) receptor modulator, which is approved as oral therapy for relapsing forms of multiple sclerosis (MS). The drug inhibits the egress of C-C chemokine receptor 7 (CCR7)-positive lymphocytes from secondary lymphatic organs including lymph nodes [4]. As a consequence, naïve and central memory T (TCM) cells are reduced in the circulation, leading to a relative increase of effector and effector memory T (TEM) cell frequencies [5]. Despite the marked reduction in the peripheral lymphocyte count the incidence of most infections is comparable to patients receiving placebo or interferon-β-1a [6,7]. Treatment with fingolimod, however, is associated with increased rates of both primary and secondary VZV infections. Two fatal cases and severe VZV infections have been reported in the pivotal trials and post-marketing setting [6,8,9,10]. The comprehensive analysis of 11915 patient years of treatment with fingolimod disclosed a nearly twice as high incidence rate of VZV infections compared to the placebo group. Post-marketing data showed a 2–3 times higher risk of VZV infection compared to other disease-modifying therapies [11]. It is, therefore, important to understand the immunological basis of why fingolimod selectively increases the risk of VZV reactivation. It may be assumed that fingolimod interferes with the function of lymphocyte subpopulations relevant for immune surveillance and memory immune responses specific for VZV in a subgroup of patients. Notably, cases of severe herpes virus infections have also been reported with other disease-modifying treatments including alemtuzumab and natalizumab.

Here, we report the time course of antibody responses against VZV and immune cell phenotypes in a patient with MS who developed herpes zoster reactivation during treatment with fingolimod. This report provides insights into the dynamics of systemic immune responses during VZV reactivation and immune reconstitution after treatment cessation and antiviral treatment.

## 2. Results and Discussion

### 2.1. Results

#### 2.1.1. Clinical Case Report

We report a 30-year-old man who presented with vesicular rash, erythema, pain and allodynia in the dermatome supplied by the first trigeminal nerve in May 2014. He had been suffering from severe holocranial headache for about one week and was taking oral valacyclovir (3.000 mg per day) and cefuroxime (1000 mg per day) since the previous day. Meningeal signs and fever were absent. The diagnosis of relapsing-remitting MS was made in April 2011 according to McDonald criteria after two relapses with dissemination in space [12]. Natalizumab treatment was initiated in September 2012 because of ongoing clinical and radiological disease activity on interferon-β-1b 8 Mio I.U. (International Units), s.c. every other day. The EDSS was 2.5 at this time. His JCV serology was negative on repeated testing (May 2012 and November 2013). Side effects in terms of fatigue and tiredness led to discontinuation of natalizumab after 16 monthly cycles (i.v., 300 mg). Fingolimod was started in March 2014 after a natalizumab wash-out period of 8 weeks (EDSS 2.5). At that time, he tested positive for serum VZV-IgG antibodies (1.700 mIU/mL). He did not report any history of recurrent VZV disease.

**Figure 1:**
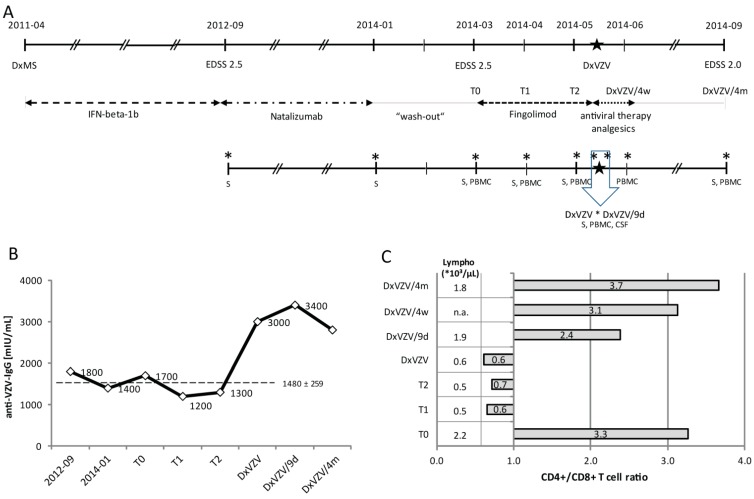
Disease course, varicella zoster virus (VZV)-specific antibody response, and lymphocyte reconstitution after fingolimod discontinuation. (**A**) Schematic overview of the clinical course, sequential treatments, sample collection time points, and sample specimen; (**B**) VZV-specific serum IgG measured over a period of two years at defined time points including start and end of natalizumab treatment, start (T0) and four (T1) and eight (T2) weeks of fingolimod treatment, diagnosis of VZV reactivation (DxVZV), and two follow-up visits nine days (DxVZV/9d) and four months (DxVZV/4m) post VZV diagnosis. The dashed line represents mean and standard deviation of measurements from before DxVZV; (**C**) Changes of absolute lymphocyte counts (*10^3^/µL) and CD4+/CD8+ T cell ratios in response to fingolimod treatment after four weeks (T1), eight (T2) weeks, and at diagnosis of VZV reactivation, and after nine days of fingolimod discontinuation and antiviral therapy (DxVZV/9d) and four weeks (DxVZV/4w) and four months (DxVZV/4m) later. Abbreviations: CSF, cerebrospinal fluid; Dx, diagnosis; EDSS, expanded disability status score; IFN, interferon; PBMC, peripheral blood mononuclear cells; S, serum; VZV, varicella zoster virus. Big asterisk: time point of diagnosis of VZV reactivation; small asterisks: time points of sample collections.

After two months of fingolimod treatment he developed symptoms of herpes zoster reactivation. Probable VZV meningitis was diagnosed on the basis of the clinical syndrome in combination with CSF findings. CSF analysis showed an increased albumin quotient (QAlb; 8.6, age-dependent normal value <6.0) and a lymphomononuclear pleocytosis of 7 cells/µL. Red blood cells (RBCs) were absent. PCR for the detection of herpes simplex virus (HSV) and VZV in the CSF was negative. Anti-VZV IgG was detected in the serum (3.000 mIU/mL) and CSF (290 mIU/mL), whereas the VZV antibody index (0.7) was negative. He tested negative for antibodies against HSV and Cytomegalovirus (CMV) and positive for anti-Epstein-Barr-Virus IgG in serum and CSF. Fingolimod was discontinued and antiviral treatment was changed to intravenous acyclovir (10 mg/kg body weight tid). Headache, pain and allodynia responded to a combination of acetaminophen and mefenamic acid. CSF re-tap 9 days later revealed persistent pleocytosis (11 cells/µL) and QAlb increase (7.7). Testing for oligoclonal bands (OCBs) was not performed. He was discharged with oral valacyclovir (3000 mg per day) for another two weeks and analgesics. At follow-up in 9/2014 he had remained clinically stable (EDSS 2.0), whereas MRI disclosed several new lesions when compared to April 2014. Treatment with dimethyl-fumarate was initiated in January 2015. An overview of the clinical course, sequential treatments, and sample collection time points is shown in Figure 1(Ae)

#### 2.1.2. Serial Analysis of Humoral and Cellular Immune Parameters

In our patient, changes in the humoral response to VZV reactivation were investigated from frozen serum samples collected on eight occasions. Anti-VZV serum IgG remained stable (mean 1.480 mIU/mL, standard deviation (SD) ± 259) over a period of almost two years until he developed zoster reactivation (Figure 1B). At this time point, the titer had increased 2.3-fold within one week (T2: 1.300 mIU/mL *versus* DxVZV: 3.000 mIU/mL), and remained increased four months later (2.800 mIU/mL). Serum IgG, serum albumin, and total serum protein assessed from the same samples were unchanged (data not shown).

**Figure 2:**
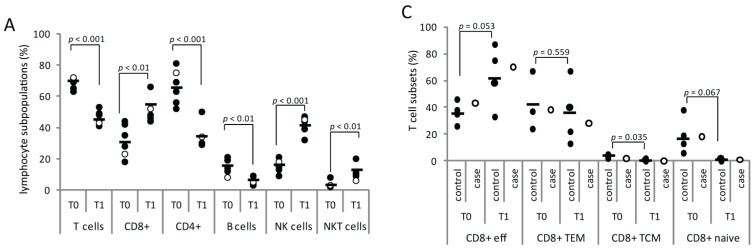

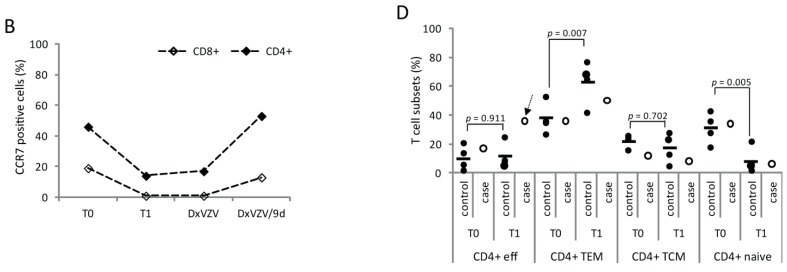
Lymphocyte frequencies and fingolimod treatment. Percentages of lymphocyte subpopulations (**A**) and T cell subsets (**C**,**D**) at initiation (T0) and after four weeks (T1) of fingolimod treatment in the peripheral blood of our case (open circle) and five other MS patients (controls, black circles); (**B**) Decrease and reconstitution of CCR7+ subsets of CD8+ (open diamond) and CD4+ (black diamond) T cells in response to fingolimod treatment (T1, DxVZV) and discontinuation (DxVZV/9d). The arrow (**D**) highlights the 3.1-fold higher effector CD4+ T cell frequencies of our case compared to controls. Bars represent mean values. Abbreviations: CCR7, C-C chemokine receptor 7; Dx, diagnosis; eff, effector cell; NK, natural killer cells; NKT, natural killer T cells; TCM, central memory T cells; TEM, effector memory T cells; VZV, varicella zoster virus.

Absolute lymphocyte numbers and the CD4+/CD8+ T cell ratio before initiation of fingolimod treatment, after four and eight weeks on fingolimod, and at diagnosis of VZV reactivation did not reveal changes other than those attributable to S1P receptor modulation (Figure 1C). After four weeks of fingolimod treatment (T1) the redistribution of major lymphocyte subsets (T-, B-, NK, and NKT cells) involved lower frequencies of T and B cells (T cells: 71% (T0)→41% (T1); B cells: 8%→4%) and an increase of NK and NKT cells (NK: 18% (T0)→45% (T1); NKT: 3%→7%). We found similar results in five other MS patients who had received fingolimod for one month after natalizumab discontinuation and a wash-out period of 8 weeks (Figure 2A).

CD4+ and CD8+ T cells were subtyped according to their expression of CD45R0 and CCR7 into naïve (CD45R0 − CCR7+), central memory (CD45R0 + CCR7+, TCM), memory effector (CD45R0 + CCR7−, TEM), and effector (CD45R0 − CCR7−) T cells. After four weeks of fingolimod treatment CCR7+ T cells were reduced in the blood (CD8+CCR7+: 19% (T0)→1% (T1); CD4+CCR7+: 46%→14%) and remained low until diagnosis of VZV reactivation (Figure 2B). T cell subtyping in four controls receiving fingolimod for four weeks showed a marked increase of effector cell frequencies in the CD8+ T cell subpopulation (T0: mean 36%, SD ± 8.3, T1: mean 63%, SD ± 23.4; *p* = 0.053) and of TEM frequencies in the CD4+ T cell population (T0: mean 38%, SD ± 10.9, T1: mean 63%, SD ± 14.9; *p* = 0.007). The fingolimod-induced T cell redistributions of our case were in line with those from controls with one exception (Figure 2C,D): the effector CD4+ T cell frequencies increased from 17% to 36% (3.1-fold higher than in controls (mean 12%, SD ± 9.0)) four weeks after initiation of fingolimod therapy.

#### 2.1.3. Cellular Immune Parameters and Immune Phenotypes of Peripheral Blood and Cerebrospinal Fluid (CSF) Cells during Intravenous Acyclovir and Fingolimod (FTY) Discontinuation

The confirmation of VZV infection was followed by prompt initiation of antiviral treatment with acyclovir and discontinuation of fingolimod. We found a reversal of S1P receptor-associated immunomodulation in the peripheral blood after a treatment discontinuation of nine days. The absolute lymphocyte count was back to 1.9 × 10^3^/µL compared to 2.2 × 10^3^/µL prior to and 0.5 × 10^3^/µL after one and two months of fingolimod treatment (Figure 1C). The lymphocyte composition had normalized (T cells: 69%, B cells: 4%, NK: 49%, NKT: 7%; CD4+/CD8+ ratio: 2.4) and CCR7+ T cells had reappeared to similar frequencies as before treatment start (Figure 2B).

**Figure 3:**
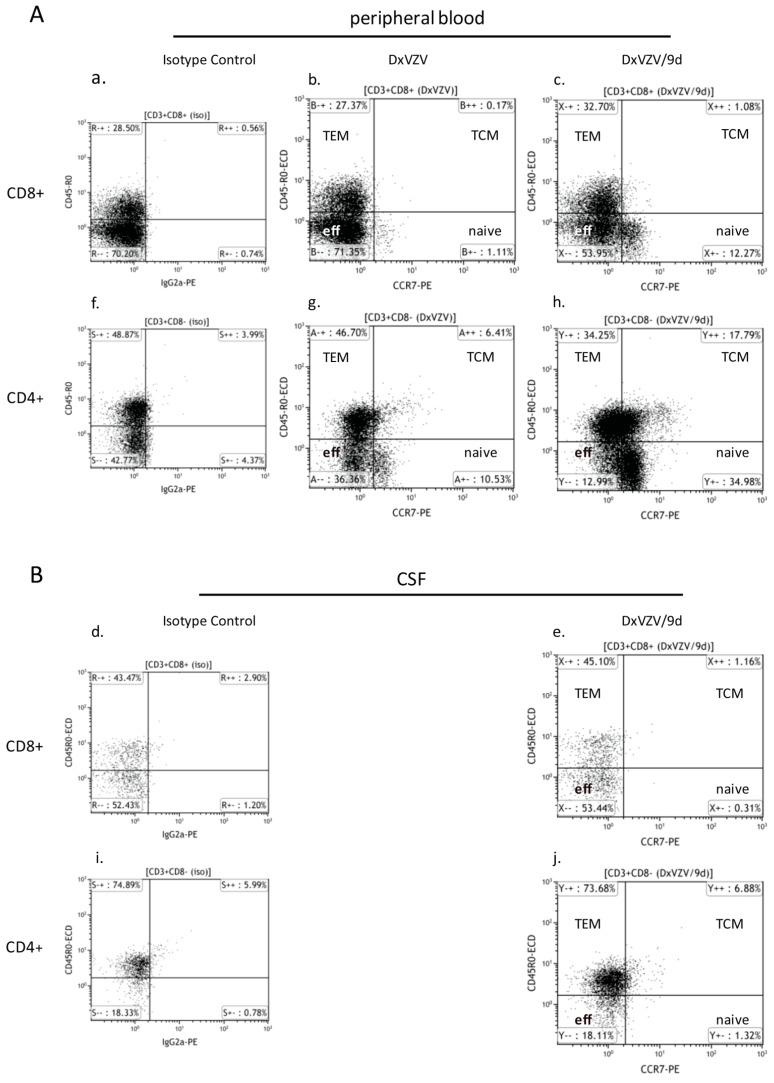
Effect of fingolimod discontinuation and CCR7 expression. The dot blots illustrate the CCR7 expression of CD8+ (**upper panel**, **a**–**e**) and CD4+ (**lower panel**, **f**–**j**) T cells in the peripheral blood (**A**) and CSF (**B**) under fingolimod treatment (DxVZV; **b** and **g**) and after nine days from fingolimod discontinuation (DxVZV/9d; peripheral blood: **c**, **h**; CSF: **e**, **j**). The corresponding isotype controls are depicted in **a**, **d**, **f** and **i**. Abbreviations: CCR7, C–C chemokine receptor 7; CSF, cerebrospinal fluid; TCM, central memory T cells; TEM, effector memory T cells.

We observed a similar phenotype of CSF lymphocytes at diagnosis of VZV reactivation and after nine days of fingolimod discontinuation. The vast majority were T cells (91% *versus* 93%) with a CD4+/CD8+ T cell ratio of 2.4 and 2.7, respectively. B cells were below 3% and NK/NKT cells below 7% at both examinations. Subtyping according to the expression of CCR7 and CD45R0 was only performed at the control tap. CSF T cells consisted exclusively of TEM and effector cells. Nine days after discontinuation of fingolimod CCR7+ T cells were not detected in the CSF which was in contrast to the findings in peripheral blood (Figure 3).

### 2.2. Discussion

Fingolimod has been shown to attenuate the antiviral immune response in MS patients as evidenced by reduced numbers of IFN-γ-producing T cells and decreased VZV-specific proliferation of CD4+ and CD8+ T cells [13]. In addition, frequencies of VZV-specific memory T cells are reportedly low and amount to only half of HSV- and to less than a tenth of CMV-specific T cell frequencies in immune adults [14,15]. Together, this may add to a disrupted defense against VZV reactivation from latency. Indeed, subclinical reactivation as determined by detection of VZV in saliva is a common consequence of fingolimod treatment [13]. Particularly the elderly and immunocompromised individuals, including patients previously treated with immunosuppressive drugs, are at risk of developing clinical complications caused by VZV reactivation. This case illustrates adaptive immune responses associated with clinically evident VZV reactivation in a fingolimod-treated MS patient. These include relative enrichment of an effector CD4+ T cell subset, an only modest systemic VZV-specific IgG response, and a strikingly fast reconstitution of absolute lymphocyte numbers and CCR7 expressing lymphocytes after discontinuation of fingolimod.

Several studies indicate that T cell immunity, in particular circulating T cells are most critical for maintaining control of latent VZV [3,16]. CD8+ effector and effector memory T cells are considered key players in the control of virus infections [17,18] and fingolimod was shown to impair CD8 T cell effector functions [19]. We did not perform activation studies to investigate VZV-specific T cell responses, and did not encounter changes or differences in the frequencies of CD8+ T cells. We found, however, an increased percentage of presumably highly-differentiated effector CD4+ T cells in our patient compared to fingolimod-treated patients without evidence of VZV reactivation. Schub *et al.* reported that VZV-specific CD4+ T cells in peripheral blood of non-fingolimod treated patients with zoster infection showed a shift in cytokine expression toward interferon γ positivity, an increase in CTLA-4 and PD-1, and a decrease in CD127 expression [20]. This is in line with other studies which disclosed a role for CD4+ T cells in the defense of herpes virus infections by executing immune responses to viral glycoproteins, such as the VZV glycoprotein E [21]. These cells apparently are abundant at low levels but may expand in case of infection or profound alterations of the immune cell composition.

A mild to moderate inhibitory effect of fingolimod on T cell-dependent and -independent antibody responses was first demonstrated in healthy volunteers [22]. A recent randomized, placebo-controlled trial evaluated immune responses against novel and recall antigens in fingolimod-treated MS patients [23]. The study confirmed that these patients in principle can mount an immune response but to a lower extent than patients receiving placebo. The threshold defining a sufficient seroprotection was set at an increase in antibody titers above 4-fold at 3 and 6 weeks. Our patient developed an about 2-fold VZV-specific IgG response and did not meet this criterion for sufficient seroprotection. Other studies, however, used less stringent criteria (>2-fold increase of antibody titers) for the definition of a protective immune response.

Another key finding of this study was that after nine days on intravenous acyclovir and withdrawal of fingolimod lymphocyte counts had normalized to almost pretreatment levels and CCR7+ T cells had reappeared in the peripheral blood. This was faster than expected according to fingolimod’s pharmacokinetic half-life data of about 7–9 days and studies on lymphocyte counts [22,24]. One possible explanation may be an accelerated fingolimod metabolism. We considered this unlikely as genetic polymorphisms associated with an altered drug metabolism of the cytochrome P450 (CYP450) enzyme 4F, by which fingolimod primarily is cleared, have not been described [25,26]. An accelerated fingolimod clearance caused by drug interactions related to acyclovir, acetaminophen, or mefenamic acid was unlikely as well as none of those compounds is listed as CYP450 enzyme inducer [27]. An alternative explanation for the fast peripheral immune reconstitution was an infection-related immunological trigger that overruled the declining impact of fingolimod on lymphocyte trapping. In fact, interferon-γ secreted from activated CD4+ T cells was shown to reduce the expression of CCR7 ligand CCL21 in lymph nodes of mice eight days post infection with viral or bacterial pathogens [28]. In the settings of such a Th1-mediated antiviral immune response declining fingolimod serum concentrations might be overruled and the balance between retention and exit signals allowing recirculation of CCR7+ lymphocytes disrupted. If so, this might have relevance concerning treatment decisions about continuing or pausing fingolimod in case of VZV infection. A rapid lymphocyte reconstitution of two to three weeks was reported by Gross and coworkers in a patient with cranial VZV polyneuritis during fingolimod treatment [8]. Follow-up MRI of their patient revealed several new lesions and a relapse after three and five months respectively after discontinuation of fingolimod, which they attributed to an immune reconstitution inflammatory syndrome (IRIS) due to the rapid restoration of immune surveillance. Similarly, follow-up MRI of our patient revealed several new brain lesions four months after cessation of fingolimod giving rise to speculations about MS-related disease *versus* IRIS. Another question is as to which extent peripheral immune reconstitution was important for containment of VZV reactivation. In contrast to the peripheral blood CCR7+ T cells were lacking in the CSF. Again, we do not know if any sustained other effect of fingolimod than S1P receptor modulation had interfered with reappearance of CCR7+ T cells in the CSF compartment, or if effector and memory effector T cells were the sole executers of the anti-VZV defense. Either way, control over the viral infection was established as evidenced by the clinical improvement.

VZV-induced disease is found most frequently within six months of initiation of fingolimod and does not increase as a function of time [11]. VZV reactivation in our case occurred eight weeks after initiation of fingolimod treatment. In this context, it needs to be clarified whether previous treatment with natalizumab might have played a role. We consider this unlikely, as the CD4+/CD8+ ratio in the CSF was almost 2.5 under fingolimod and thus even higher than the mean rates reported in a previous study [29]. Alternatively, latency of a highly neuroinvasive VZV strain could have been the cause of reactivation following fingolimod treatment. Indeed, recent studies implicate differences in viral neurotropism and polyclonal immune responses against VZV related to different antigens, as well as genetic susceptibility for VZV reactivation [30,31,32]. Treatment-associated increases of T regulatory cell (Treg) numbers [33,34], enhanced functional activity of Tregs [35], and reduced numbers particularly of VZV-specific T cells in combination of steroid treatment [36] are further factors potentially implicated in facilitating VZV reactivation in fingolimod-treated patients.

Reasons why we were not able to detect VZV DNA in the CSF of our patient are likely related to the short duration of clinical symptoms, the initiation of high-dose valacyclovir one day prior to the spinal tap, and a sensitivity of VZV PCR of only 80% in immunosuppressed individuals [37]. In regard to the minor extent of CSF pleocytosis, it needs to be taken into account that fingolimod efficiently sequesters immune cells from the CNS, and normalizes cell counts in CSF [29]. Fingolimod has limited impact on intrathecal IgG synthesis or antigen-specific adaptive immune responses [22,29,34]. Lack of intrathecal but rise of peripheral VZV-specific IgG at diagnosis thus may reflect an early stage of infection.

## 3. Experimental Section

Measurements were performed from fresh peripheral blood mononuclear cells (PBMC) before fingolimod treatment was initiated (T0), after four (T1) and eight (T2) weeks of fingolimod treatment, at diagnosis of VZV reactivation (DxVZV), and nine days (DxVZV/9d), four weeks (DxVZV/4w), and four months (DxVZV/4m) post diagnosis of VZV infection. Time points of CSF analysis were DxVZV and DxVZV/9d.

For flow cytometry, PBMC were enriched by density centrifugation from venous blood collected in cell preparation tubes (CPT, Becton Dickinson AG, Basel, Switzerland). PBMC were stained for 30 min on ice with saturating amounts of antibodies in phosphate buffered saline (PBS) supplemented with 2.5% fetal calf serum. CSF samples were stained with identical concentrations of antibodies. The antibody panel included FITC-labeled anti-CD45 (J33); PE-labeled anti-CD56 (NKH-1) and anti-CCR7 (3D12; eBioscience, Vienna, Austria); ECD-labeled anti-CD3 (UCTH1) and anti-CD45R0 (UCHL1); PC5-labeled anti-CD8 (B9.11); and PC7-labeled anti-CD3 (UCTH1) and anti-CD19 (J3-119). Isotype controls were PE-labeled rat IgG2a (eBR2a, eBioscience) and ECD-labeled IgG1 (P3.6.2.8.1). Unless otherwise specified, all antibodies were purchased from Beckman Coulter (Vienna, Austria). Natural Killer (NK) T cells were identified as CD56+CD3+ lymphocytes and CD4+ T cells as CD3+CD8− T cells. Cells were acquired on a Cytometrics FC500 and analyzed using the Kaluza software (version 1.3; all Beckman Coulter).

Results of our patient were compared at T0, T1, and T2 with those of four to five other fingolimod-treated MS patients (mean age 41 years; range 33–59) who had discontinued natalizumab treatment after mean 51 months (range 29–71) because of a positive JC virus (JCV) serostatus (controls). All patients participated in a local scientific study for monitoring immunological changes in MS (ethics committee of Salzburg 415-E/1612/2-2013) and gave written informed consent for publication of results.

## 4. Conclusions

Our findings show that VZV reactivation in fingolimod-treated patients is associated with a low level systemic VZV-specific antibody response and increased number of effector CD4+ T cells. The immune reconstitution upon fingolimod discontinuation was unexpectedly fast and may have been driven by infection-related triggers counteracting lymph node retention signals. If so, this is of relevance concerning treatment decisions for cessation of fingolimod in case of VZV infection. However, whether these findings from a single case can be generalized remains unclear. Further research into fingolimod-associated VZV reactivation and immune reconstitution dynamics are mandatory to prevent morbidity and mortality associated with this potentially life-threatening condition.
